# Moiré Superstructure and Dimensional Crossover of 2D Electronic States on Nanoscale Lead Quantum Films

**DOI:** 10.1038/s41598-017-12851-0

**Published:** 2017-10-06

**Authors:** Hyo Sung Kim, Gyeongcheol Gye, Sung-Hoon Lee, Lihai Wang, Sang-Wook Cheong, Han Woong Yeom

**Affiliations:** 1 Center for Artificial Low Dimensional Electronic Systems, Institute for Basic Science (IBS), Pohang, 790-784 Korea; 2 Department of Physics, Pohang University of Science and Technology, Pohang, 790-784 Korea; 3 Laboratory for Pohang Emergent Materials, Pohang University of Science and Technology, Pohang, 790-784 Korea; 4 Rutgers Center for Emergent Materials and Department of Physics and Astronomy, Piscataway, New Jersey 08854 USA

## Abstract

We investigate using scanning tunneling microscopy and spectroscopy electronic aspects of Moiré superstructures in nanoscale Pb quantum films grown on IrTe_2_, which is a unique layered material with charge-order transitions into stripe phases. Pb ultrathin films exhibit a Moiré superstructure due to the lattice mismatch of Pb and IrTe_2_, which produces strong lateral electronic modulation of hexagonal symmetry and discreet subbands. Moreover, strongly anisotropic or 1D electronic states are formed in Pb films as modulated by the stripe charge order. Present results indicate the controllability of lateral electronic structures of various ultrathin films by extra interfacial potentials due not only to Moiré superstructures but also to novel electronic orderings of substrates.

## Introduction

Graphene and other two dimensional (2D) atomic layers have been one of the most intensively investigated topics in materials physics and engineering for a decade^1–3^. Beyond exploiting properties of single isolated atomic layers, assembling different atomic layers through the van der Waals interaction is expected to reveal new electronic properties and functionality^4^. Indeed, van der Waals interfaces of graphene with *h*-BN and graphene itself produced interesting electronic states such as cloned mini Dirac bands^5–8^ and Hofstadter states^9,10^. The key underlying physics is within extra 2D periodic potentials provided by highly ordered Moiré superstructures due to interfacial lattice mismatches^11^. However, so far the successful demonstration of 2D *electronic* modulations by Moiré superstructures has been limited to graphene cases and a very recent one for a semiconducting single-layer transition metal dichalchogenide heterostructure^12^, leaving a great room of development given the huge variety of 2D materials.

In this work, we demonstrate the modulation of 2D electronic structures of ultrathin (a few atomic layers) metal Pb films by the Moiré superstructure of a van der Waals heterointerface with a layered transition metal dichalcogenide, IrTe_2_. 2D quantum well states (QWS’s) of Pb films are found to be modulated laterally by the Moiré superstructure at the interface. Furthermore, the substrate features a transition into charge-ordered ground states^13–24^ with emergent superconductivity^25–28^. The stripe charge ordering and the lattice distortion accompanied provide an extra tunability into the 2D electronic states, that is, the 2D-1D dimensional crossover of electronic states. This work thus extends the scope of van der Waals electronic engineering in both materials and concept.

**Figure 1:**
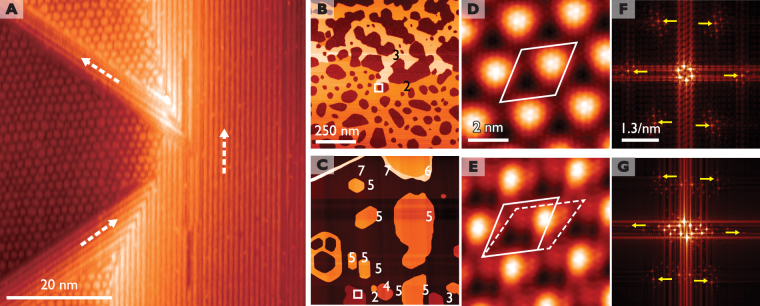
STM topography of Pb islands on top of a cleaved IrTe_2_ substrate at 4.3 K. (**A**) A typical cleaved IrTe_2_ surface shows hexagonal (H) and stripe (S) domains coexisting. (**B**) 2 ML and 3 ML Pb films on hexagonal domains. (**C**) Pb islands with different heights on a wide stripe domain. The thickness of each Pb island is indicated where a single layer height is about 0.3 nm. (**D**) and (**E**) Different Moiré superstructures are shown on the atomically resolved Pb islands with their unitcells marked. Tunneling current and bias are 1 nA and 20 mV, respectively. (**F**) and (**G**) The FFT of the STM images in (**D**) and (**E**), respectively. The 1×1 Bragg features are indicated by arrows and the other spots are the satellites due to Moiré superstructures.

Figure 1A shows the Te layer exposed after cleaving a IrTe_2_ crystal. Below the transition temperature of 260~280 K coexist the stripe phases (S) of three degenerate orientations and a distinct hexagonal phase (H) [Fig. S1a]. Their origins are commonly the charge ordering of Ir 5*d* ^3+^ and 5*d* ^4+^, which accompanies substantial lattice distortions through the Ir dimerization. The dominant phase is the stripe phase of a 5a_0_ period, which is mixed with other stripes of a 3a_0_ or 8a_0_ period as reported previously^13–24^. On the other hand, the minor phase of a hexagonal pattern has a period of about 7.4a_0_ and falls into a superconducting state below 3.1 K^29^.

When Pb atoms are deposited on to IrTe_2_, well ordered flat 2D islands of Pb(111) are formed, which are very similar to the growth on other substrates^30,31^. For low temperature growth, flat Pb(111) islands with different thickness are formed together, where electronic states of various films are accessible simultaneously and conveniently. The growth mode depends drastically on different charge orders of the substrate. While flat islands of different heights are formed with a size of a few tens of nanometers on wide stripe domains, larger connected islands of two or three monolayer (ML) thickness preferentially grow on hexagonal domains (see Fig. 1B,C and Fig. S1 in Supporting information).

Figure 1D,E shows 2 ML Pb islands on a part of the surface with stripe and hexagonal domains coexisting. Moiré patterns are observed in Pb layers on top of both domains. Pb islands on the hexagonal domain (hereafter H-Pb islands) exhibit a regular and isotropic Moiré structure [Fig. 1D and center of the Fig. S1a] but a less regular pattern of a marginally different periodicity and orientation is observed on the stripe domain (hereafter S-Pb islands) [Fig. 1E]. The fast Fourier transformation of the STM images in Fig. 1F,G indicate that the Moiré superstructure on H-Pb (S-Pb) islands has a periodicity of 2.61 nm (2.73 nm), which corresponds to a Moiré angle of 19 ± 2 (23 ± 2) degree due basically to a 11% lattice mismatch between Pb(111) and IrTe_2_ (see Fig. S2 in Supporting information). The small difference between H-Pb and S-Pb islands is due to the extra strain in the hexagonal phase^29^. On S-Pb islands, the Moiré structure is mixed with an additional stripe modulation of the same periodicity and orientation with the underlying stripe order (Fig. S1e in Supporting information). On the other hand, the hexagonal charge order pattern overlaps very closely with the Moiré superstructure leaving no noticeable footprint of its own on Pb islands.

**Figure 2:**
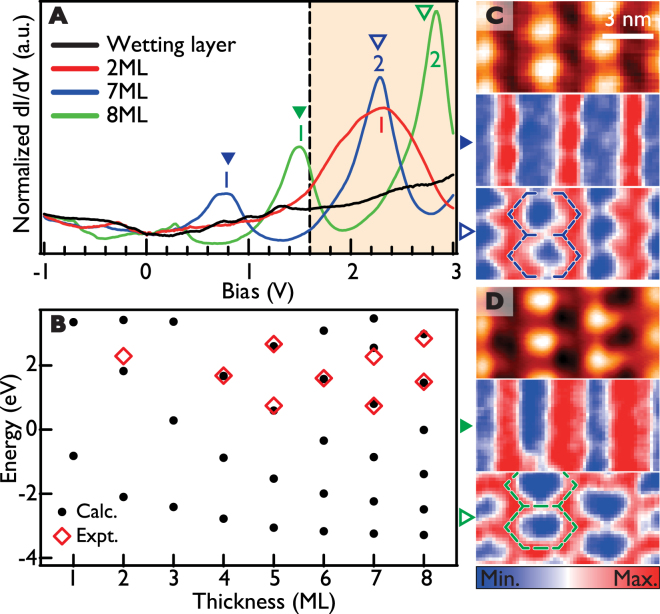
QWS’s of Pb islands on the stripe domain of IrTe_2_ in *dI/dV* spectra and maps. (**A**) STS (*dI/dV*) spectra of the wetting layer, 2, 7 and 8 ML of Pb films, respectively. (**B**) Calculated Pb quantum well state energies, sampled at the center of the Brillouin zone, and corresponding experimental values are given by black dots and red rectangles. (**C**) and (**D**) STM topography and the LDOS maps of the first (middle) and second (bottom) quantum well states of 7 ML and 8 ML Pb films, respectively. The energies for the LDOS maps are marked by solid and empty triangles in (**A**). The LDOS maps indicates dimensional crossover around 1.6 eV.

**Figure 3:**
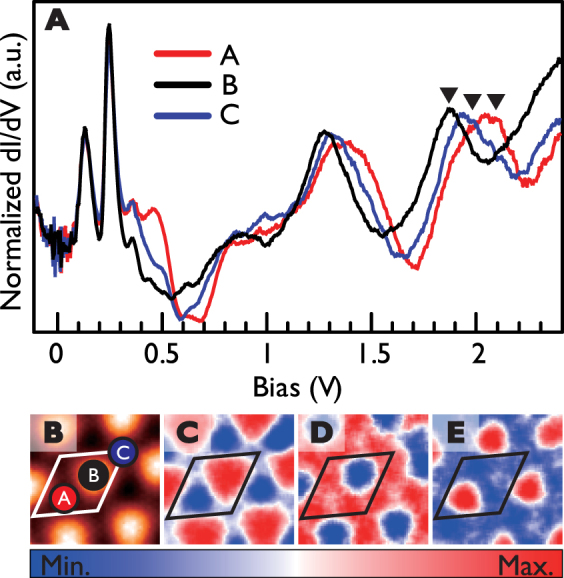
*dI/dV* spectra and maps of 2 ML Pb islands on the hexagonal domain of IrTe_2_. (**A**) Averaged STS (*dI/dV*) spectra from three different regions on the H-Pb film. (**B**) STM topography for the comparison. (**C**–**E**) *dI/dV* LDOS maps of the split 1st quantum well states at 1.86, 1.98 and 2.1 eV, respectively. The other spectral features at lower energies are mainly due to in-plane *p*-orbitals of the Pb film.

Electronic states within well ordered Pb films are quantized vertically to form 1D QWS’s, which are well defined 2D electronic states laterally^30–33^. These QWS’s are well resolved in STS (tunneling *dI/dV*) spectra for empty states as shown in Fig. 2A. In filled state, QWS’s are observed only at very low energies near the Fermi energy^30–33^. The QWS energies agree well with our own density functional theory (DFT) calculations for floating films [Fig. 2B] and the previous works^30–33^. We map local density of states (LDOS) of each QWS laterally to reveal the effect of Moiré superstructures. The QWS LDOS exhibits strong lateral modulations as shown in Fig. 2C,D, whose periodicity follows exactly that of the Moiré superstructure. Very intriguingly, the LDOS of the first QWS above Fermi level for the S-Pb films shows a linear pattern indicating 1D electronic states laterally, which will be discussed later. All LDOS modulations are due to the small energy splitting or shift of electronic states between dark and bright LDOS regions, which varies between 10 and 300 meV with a tendency to increase for a thinner film and for a QWS closer to the Fermi energy (see Fig. 3 and Fig. S3 in Supporting information). As shown by lower energy spectral features without energy shifts in Fig. 3A, the QWS energy change cannot be explained by any overall energy shift such as the local work function change.

The QWS energy splitting is thus most clearly seen for the thinnest film, 2 ML, especially on H-Pb films where the hexagonal Moiré superstructure is better ordered (see Fig. S4 in Supporting information). As shown in Fig. 3, the QWS’s are split into three subbands with distinct LDOS lateral patterns and the energy splitting amounts to 120 meV. The split states have distinct LDOS patterns, which are located on the high symmetry sites A (Fig. 3E) and B (Fig. 3C) or split around A/B (Fig. 3D). The LDOS patterns of the states on A and B sites are essentially the same as those of the Moiré superstructure of graphene with Dirac subbands^8^. While less resolved, consistent energy splittings can also be found on Pb films on stripe domains. We argue that the periodic lateral potential provided by the Moiré superstructure is strong enough to split each QWS into subbands and this effect gets stronger for thinner films and closer to Fermi level.

**Figure 4:**
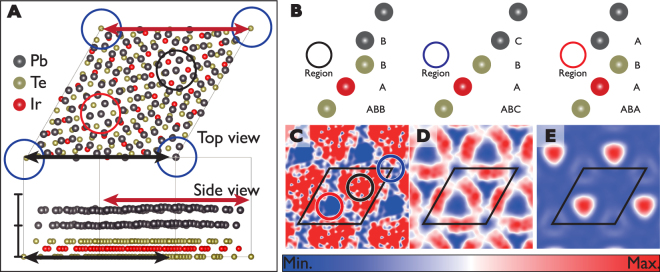
Calculated electronic structures of Pb islands on the hexagonal domain. (**A**) Atomic scale model structure for H-Pb phase. Black, blue and red circles in diamond unitcell correspond to the region of the A, B and C. Heights distribution of 2 ML Pb film is shown. The distance of upper and lower Te layers is about 0.28 nm. The 1 nm vertical scale bar is shown for eyes. The maximum vertical corrugation in this calculation is about 0.07 nm, which is about half of the experimental value. (**B**) The stacking mode of each site within a Moiré unitcell. (**C–E**) Calculated *dI/dV* maps of the 1st quantum well state at 2.14, 2.28 and 2.38 eV. Color scale spans 17%, 78% and 100% of LDOS maxima in (**C**), (**D**) and (**E**), respectively.

This conclusion is corroborated by DFT calculations. Figure 4A is an atomic structure of a unitcell of the 2 ML Pb film on top of a single layer IrTe_2_. In this model, the supercell size is 2.74 nm and the Moiré angle is 22.9 degree, which are quite well matched with the experiment. The van der Waals contribution is calculated to be about 65% of the whole interfacial energy gain. One can see that the film is corrugated, that is, strained, due to the Moiré potential and A, B and C sites have distinct stacking registries with the substrate [Fig. 4B]. The vertical corrugation with B sites protruded is consistent with the STM topography (Fig. 3A) where those sites are brighter. The calculated DOS indicates the complex energy splitting of the QWS which mainly comes from the *P*_z_ orbital of Pb (see Fig. S5 in Supporting information). We compared the fully relaxed structure shown in Fig. 4A and also the Pb film of the same strained structure but the IrTe_2_ layer detached. The size of energy splittings and the LDOS patterns of the split QWS are better reproduced in the latter structure. The calculated LDOS patterns are shown in Fig. 4. This suggests that the QWS splitting in the present case is more closely related to the periodic structural distortion of the film than the direct electronic coupling with the substrate. The effect of the lateral strain on a QWS has been well known in compound semiconductor multilayer structures^34,35^. Note that the present structure model for the IrTe_2_ layer is so simple that the charge ordering and its complex electronic structure are not included at all. This limitation makes it difficult to account for the electronic coupling effect accurately. Nevertheless, it is clear that the Moiré superstructure, the periodically corrugated film structure, induces strong electronic modulation on the Pb film.

While QWS’s and the Moiré superstructure Pb films have been extensively investigated^11,30–33,36^, only few works reported the lateral LDOS modulation of QWS following the Moiré superstructure^31^. These modulations were observed in the intensity^31^ and energy^36^ of a QWS peak, which were qualitatively attributed to the lateral variation of the electron screening or the interfacial phase shift^31^, respectively^36^, without any theoretical analysis. The electron screening to reduce the QWS *intensity* is not related to the energy splitting observed here. Moreover, the gradual lateral variation of the interfacial phase shift is not compatible with the energy splitting of a QWS (Fig. S4 Supporting information). Note also that the interfacial phase shift is an oversimplified model, which assumes the film structure and band structure undistorted in contrast to the present experimental and calculated results.

The present case is one of very few demonstrations of the Moiré modulation of 2D electronic states beyond the single layer graphene cases^5–10^ and can be applied to various ultrathin films of metal, semiconductors, and insulators since these films would have QWS 2D bands when sufficiently thin. To the best of our knowledge, the only other case is a very recent one for a semiconducting single-layer transition metal dichalchogenide heterostructure^12^. The observed electronic modulation therein is remarkably similar to the present result. This work didn’t try to model the whole Moiré supercell as performed here but instead used simpler epitaxial structures with different interlayer registries, mimicking A, B, and C sites, separately. Therefore, while the strain of the film cannot easily be addressed, the electronic interlayer coupling was discussed in detail. We, thus, believe that this and the present results are complimentary to grasp the whole mechanism of the Moiré modulation of 2D electronic states, indicating both the importance of the interlayer stacking registry and the strain of the film.

On the other hand, the STM topography shows not only the Moiré superstructure but also an extra modulation due to a stripe charge order pattern for S-Pb films [Figs 1 and 2]. When the QWS energy becomes closer to the Fermi energy than 1.6 eV, the hexagonal (honeycomb) LDOS pattern evolves gradually into 1D stripe patterns (see also Fig. S2). The stripe LDOS pattern is related to the QWS energy splitting of about 60–140 meV (see Fig. S3 in Supporting information). This indicates the presence of a stripe potential at the interface, which competes with the hexagonal Moiré potential becomes dominating at a low energy. The 1D or uniaxial interfacial potential can be provided by the stripe charge order of the substrate which induces a substantial uniaxial lattice corrugation (about 10% vertically) and a strong charge modulation on the surface Te layer^23^. The formation of a 1D Moiré superstructure and 1D electronic states under uniaxial strain in a van der Waals interfaces was theoretically suggested very recently^37^. Probing further the 1D nature of the uniaxially strained QWS would be very interesting but out of the scope of the present work. The present result suggests that an emergent electronic superstructure of the substrate can offer an extra knob into the 2D electronic engineering at van der Waals heterointerfaces beyond the lattice mismatch. The proximity coupling of exotic electronic states of the substrate with those of the overlayer film may be expected at such a ‘complex’ heterointerface.

## Methods

### Sample preparation

Single crystals of IrTe_2_ were grown by Te flux using pre-sintered IrTe_2_ polycrystals as reported previously^19^. Samples are cleaved in a vacuum better than 5 × 10^−10^ torr at room temperature or 86 K, lower than the transition temperature. Pb is grown at 86 K or room temperature. In the case of low temperature deposition, we perform an annealing to room temperature for 12–16 Hour.

### STM measurement

All the STM measurements were obtained with a commercial ultrahigh vacuum cryogenic STM (Specs, Germany) in the constant-current mode with PtIr tips at 4.3 K. The differential conductance, *dI/dV*, was measured using the lock-in detection with a modulation of 1.17 kHz.

### DFT calculations

Density functional theory calculations were performed using the Vienna Ab initio Simulation Package^38^. The projector augmented-wave potentials^39^ with the Perdew, Burke, and Ernzerhof exchange-correlation energy^40^ and the DFT-D3(BJ) method^41,42^ were used to simulate the system. A lattice constant of the supercell is 27.375 Å and a vacuum region is almost 10 Å. ($\sqrt{63}\times \sqrt{63}$) Pb (111) bilayers are on the (7 × 7) 1*T*-IrTe_2_ monolayer with a twisted angle 2.7 degree. The atoms of the slab system were fully relaxed except lower telluriums fixed to represent the bulk property of the 1*T*-IrTe_2_ substrate. The plane wave cut-off energy was set to 210.86 eV and the Monkhorst-Pack k-point mesh was 3 × 3 × 1.

## Electronic supplementary material

Supplementary Information
