# Supplementary material for: Moiré Superstructure and Dimensional Crossover of 2D Electronic States on Nanoscale Lead Quantum Films
Source: Sci Rep. 2017 Oct 6;7:12735. doi: 10.1038/s41598-017-12851-0 (PMC5630570; doi:10.1038/s41598-017-12851-0)
Supplement: Supplementary file 1 — Supplementary Information [file 41598_2017_12851_MOESM1_ESM.pdf]

Supplementary Materials for

Moiré Superstructure and Dimensional Crossover of 2D Electronic States on Nanoscale Lead Quantum Films

Hyo Sung Kim[1,2], Gyeongcheol Gye[1,2], Sung-Hoon Lee[1,2], Lihai Wang[3], Sang-Wook Cheong[2,3,4], Han Woong Yeom[1,2*]

[1]*Center for Artificial Low Dimensional Electronic Systems, Institute for Basic Science (IBS), Pohang 790-784, Korea*

[2]*Department of Physics, Pohang University of Science and Technology, Pohang 790-784, Korea*

[3]*Laboratory for Pohang Emergent Materials, Pohang University of Science and Technology, Pohang 790-784, Korea*

[4]*Rutgers Center for Emergent Materials and Department of Physics and Astronomy, Piscataway, New Jersey 08854, USA*

*To whom all correspondence should be addressed; yeom@postech.ac.kr.

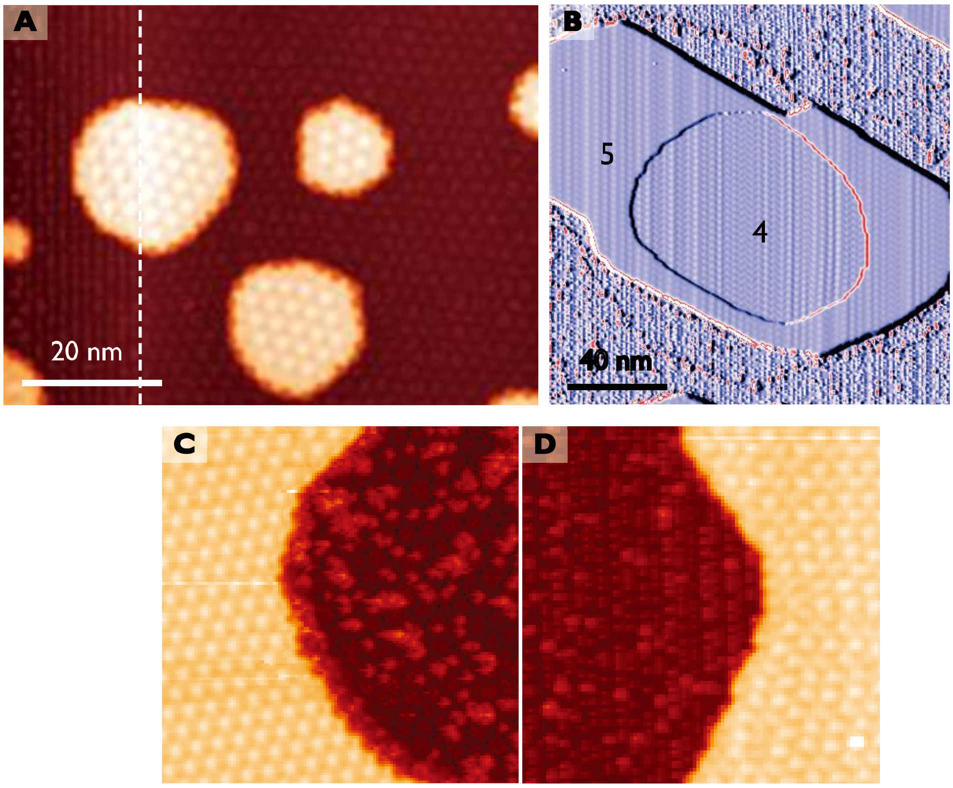

Supplementary Figure 1 │ 2 ML Pb islands on hexagonal and stripe phases of $IrTe_2$ at 4.3 K. (a) Pb islands on the hexagonal and stripe phase. **(b)** Derivated STM image of Pb 4 and 5 ML islands with stripe phase. The modulatipon of the Moiré pattern follows a stripe charge order direction. **(c), (d)** Enlarged STM images of the squared areas in Fig. 1 **(b)** and **(c).** In Pb islands on the hexagonal phase (H-Pb), a uniform superlattice is shown, a similar but less regular Moiré pattern is formed on in those on the stripe phase (S-Pb). The total amount of Pb deposited is 1.2 ML.

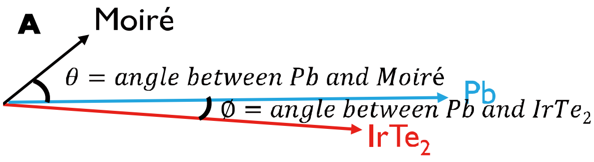

$$\lambda = \frac{(1+\delta)a}{\sqrt{2(1+\delta)(1-cos\phi)+\delta^2}}$$

$$tan\theta = \frac{-sin\phi}{(1+\delta)-cos\phi}$$

B	λ (nm)	θ (°)	ϕ (°)	a_0 (nm)
Calc.	2.93 nm	22.9	-3	0.393
S-Pb	2.73 nm	23±2	-3	0.393
H-Pb	2.61 nm	19±2	-3	0.403

Supplementary Figure 2 │ Calculated and experimental values of the Moiré wave length and angle between Moiré, Pb and $IrTe_2$ lattice. (a) Angle relation for the Moiré superstructure and $IrTe_2$ lattice with respect to the Pb lattice direction, and algebra to obtain the wave length and angle (ref. 6). Lattice mismatch delta (δ) defines as a ratio between $IrTe_2$ and Pb lattice. **(b)** Results of the calculated value where the lattice distortion is not included, and of the experimental value on the stripe and hexagonal phases. The value of a_0 means a lattice constant of the theory in calculation case, and the calculated value from the Moiré wave length (λ), theta (θ) and phi (ϕ) in S-Pb and H-Pb case.

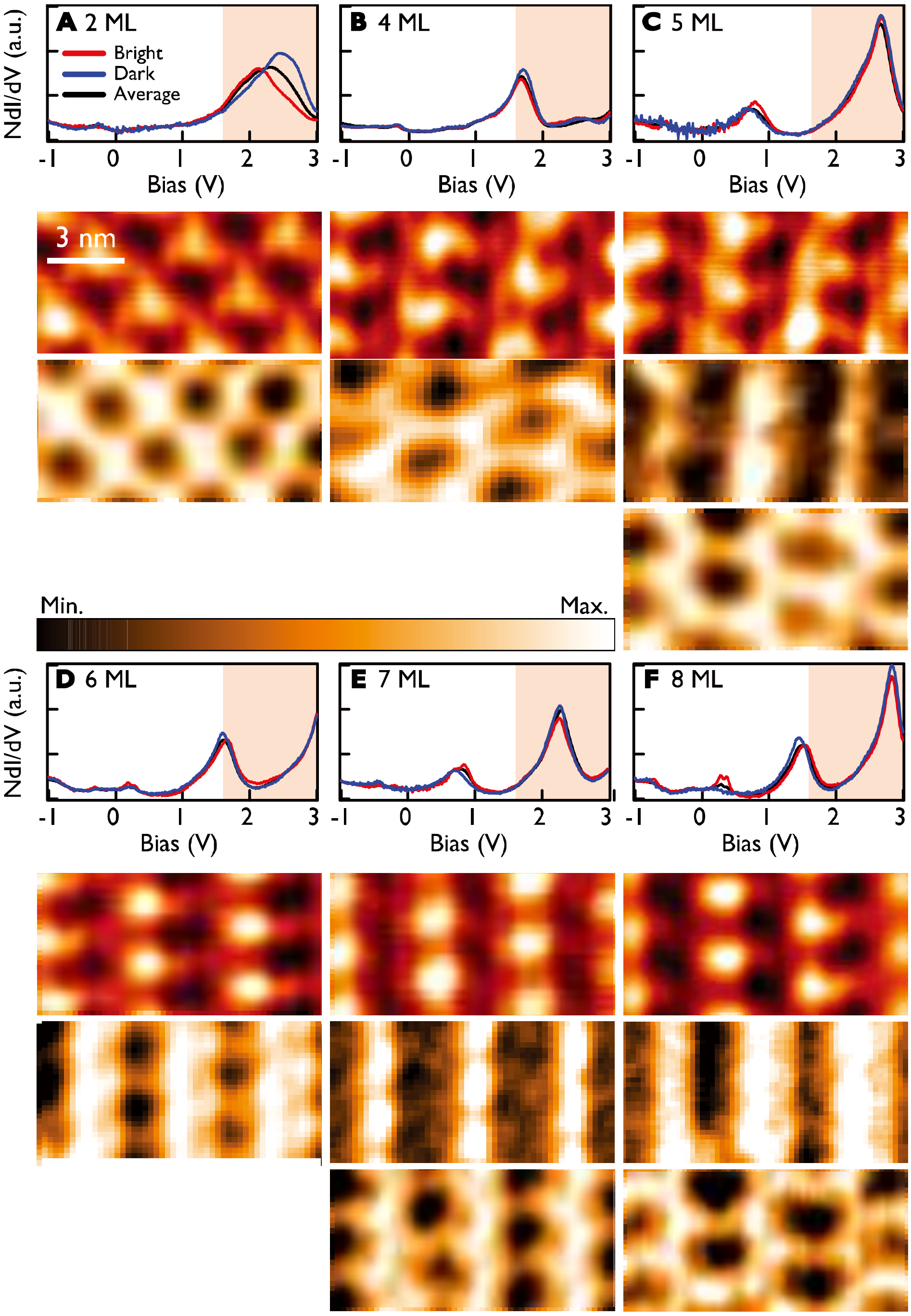

Supplementary Figure 3 │ STM topography and *dI/dV* maps for quantum well states of various Pb films on the stripe phase (S-Pb of $IrTe_2$). (a)-(f) An extended set of LDOS maps of S-Pb islands. First rows are STS curves of bright and dark regions and their average of the STM topography images shown in second rows. STM tunneling conditions are V_s=1 V and I_t=100 pA. Third and fourth rows correspond to *dI/dV* LDOS maps of the first and second quantum well states, respectively. Energy splittings between bright and dark regions are, 0.345

eV in 2 ML, 0.03 eV in 4 ML, 0.125 and 0.016 eV (the first and the second QWS's) in 5 ML, 0.062 eV in 6 ML, 0.14 and 0 eV (the first and the second QWS's) in 7 ML and 0.078 and 0.015 eV (the first and the second QWS's) in 8 ML, respectively.

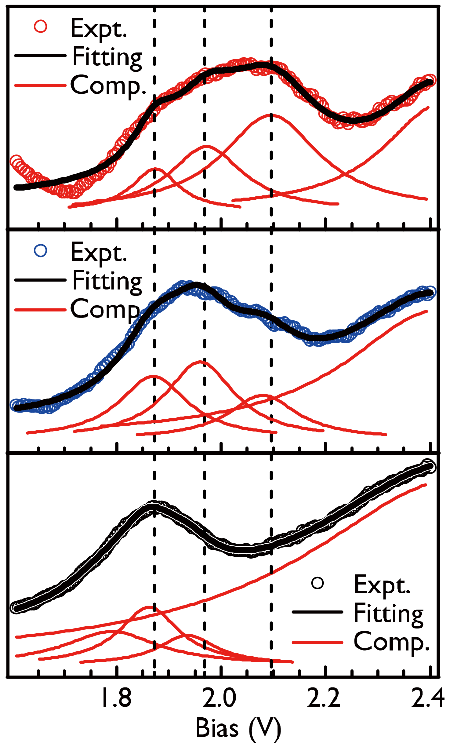

Supplementary Figure 4 | Curve fittings of the first QWS. Red, blue and black curves correspond to A, C and B regions in unit cell. Each QWS peaks are splitted by three components which amplitude varies depending on the location in supercell. Colored circles, black and red solid lines are correspond to experimental data, fitting curves and components in fitting curves.

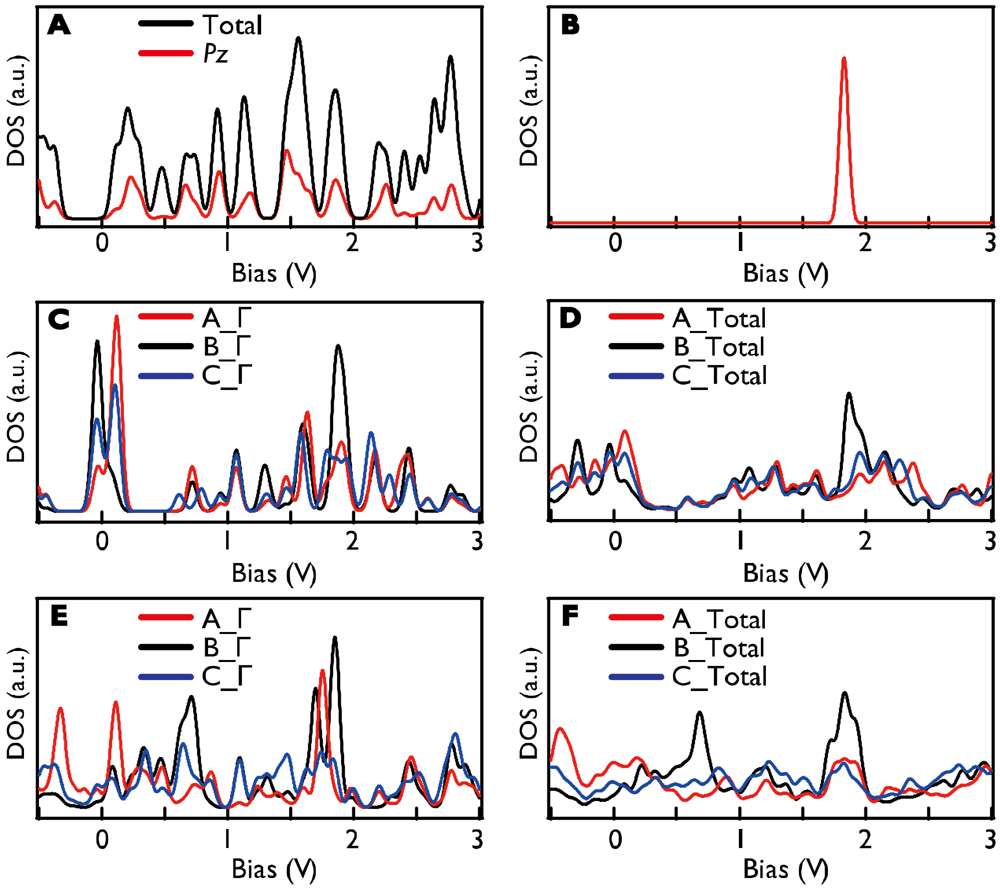

Supplementary Figure 5 │ Calculated density of states of 2 ML Pb films on $IrTe_2$. (a) Total and partial density of states of Pb bilayer over the whole Brillouin zone. (b) QWS on Pb bilayer near the gamma point. (c), (d) Partial LDOS contribution (P_z) without the substrate on A, B, and C sites of a Moiré supercell of a 2 ML Pb film. Near the gamma point and over the whole Brillouin zone, respectively. The structural strain effect is included. (e), (f) Similar calculations to (c), (d) but for a 2 ML Pb film with the substrate. Near the gamma point and over the whole Brillouin zone, respectively.
